# Congenital protein C deficiency and thrombosis in a dog

**DOI:** 10.1111/jvim.15766

**Published:** 2020-04-11

**Authors:** Darren Kelly, Florence Juvet, Gary Moore

**Affiliations:** ^1^ Southern Counties Veterinary Specialists, Forest Corner Farm Hampshire UK; ^2^ Viapath London UK

**Keywords:** antiphospholipid, clopidogrel, rivaroxaban, thromboembolism

## Abstract

Congenital protein C deficiency is an important cause of thrombosis in humans but is not described in dogs. A 4‐year‐old Hungarian Vizsla was presented for investigation of acute onset of ascites. Computed tomography of the chest and abdomen and echocardiography confirmed a large thrombus within the right ventricle. A cause for thrombosis was not initially identified. The clinical signs resolved rapidly and the dog was administered clopidogrel and discharged. Plasma protein C activity measured 2 and 6 weeks later was markedly lower than expected on both occasions. All known causes of acquired protein C deficiency were excluded, and the dog was diagnosed with a congenital protein C deficiency. After diagnosis, the administration of clopidogrel was stopped and administration of rivaroxaban was started. The dog remains well with no evidence of recurrent thrombosis with 6 months of follow‐up.

**Abbreviations**
CT: computed tomography
DOAC: direct oral anticoagulant

## 1 INTRODUCTION

Protein C is a circulating, vitamin K‐dependent protease produced by the liver with importance in the regulation of coagulation. The activated form of protein C acts as an anticoagulant by inactivating factors Va and VIIIa and thereby regulating the subsequent thrombin generation. Consequently, deficiencies of this protein predispose to venous thrombotic events. Deficiencies in protein C can be hereditary or acquired. While severe congenital deficiencies in people are associated with clinical signs during the neonatal period, mild deficiencies can be asymptomatic or result in recurrent episodes of venous thrombosis later in life.^1^ A single case report of congenital protein C deficiency in animals describes the disease in a thoroughbred foal.^2^ Acquired protein C deficiency can occur in people because of hepatic dysfunction, kidney disease, administration or intoxication with vitamin K antagonists, sepsis, and disseminated intravascular coagulopathy.^1^ Acquired protein C deficiencies in animals occur in association with liver failure and congenital portosystemic shunts, sepsis, acute inflammatory states, and disseminated intravascular coagulopathy.^3^, ^4^, ^5^, ^6^ This is a case of a congenital deficiency of protein C in a dog, causing unprovoked thrombosis.

## 2 CASE HISTORY AND DIAGNOSTIC FINDINGS

A 4‐year, 9‐month‐old male neutered Hungarian Vizsla was referred for investigation of a 2‐3 day history of acute onset abdominal enlargement and SC edema. The owner reported acute onset of clinical signs, which had initially progressed over the first 48 hours. Abdominal effusion was documented during ultrasound examination at the referring practice. The dog was reported to be otherwise well with no previous medical problems.

At the time of presentation, the dog was bright and alert. On physical examination, the abdomen was distended and there was localized SC edema affecting the caudoventral abdominal wall and inguinal region. No other abnormalities were detected on physical examination.

No abnormalities were detected on initial hematology. Biochemistry showed only mild‐moderate elevation in serum alanine aminotransferase (288 U/L; reference range 0.0‐25.0 U/L) activity. Postprandial bile acids were within reference range (3.3 mg/L; reference range 0.0‐9.8 mg/L). No abnormalities were detected on full urine analysis. Urine protein : creatinine ratio was 0.15 (reference range 0.0‐1.0), and urine specific gravity was 1.040. Prothrombin and activated‐partial thromboplastin times were within reference ranges. Plasma fibrinogen concentration was slightly below reference range on the day of presentation (1.96 g/L; reference range 2.0‐4.0 g/L) but was found to be normal on the day after presentation (3.33 g/L). d‐dimer concentrations were elevated on the day of presentation (>250 to <500 ng/mL; reference range <250 ng/mL) and the day after presentation (>500 to <1000 ng/mL)

An echocardiogram was performed by a board‐certified specialist in veterinary cardiology and confirmed the presence of a space‐occupying lesion within the right ventricle, with part of the lesion extending into the right atrium during systole. Left ventricular end‐diastolic and systolic diameters were normal with normal systolic function and normal left atrial size. Right and left ventricular outflow velocities were normal, and there was no evidence of pulmonary hypertension or elevated right ventricular pressure.

Computed tomographic (CT) imaging of the thoracic, abdominal, and pelvic cavities was performed under general anesthesia, precontrast and postcontrast (Omnipaque [iohexol 300 mg/mL], GE Healthcare, New York, New York) along with an ECG‐gated cardiac CT. The images were reviewed by a board‐certified specialist in veterinary diagnostic imaging. A lobulated, approximately 2‐cm diameter mass‐lesion causing a filling defect within the contrast pool of the right ventricle was seen. The heart appeared otherwise unremarkable. A moderate‐large volume of abdominal free‐fluid was seen along with SC fluid accumulation affecting the ventral abdominal and inguinal regions. No other relevant changes were seen and all visualized vascular structures appeared normal.

Analysis of the abdominal fluid (collected by abdominocentesis and performed before imaging) confirmed a protein‐rich transudate (total protein 40 g/L, albumin 19 g/L, globulin 21 g/L, albumin : globulin ratio 0.90). Nucleated cell count was low (2.78/μL) and consisted of mixed neutrophils, lymphocytes, macrophages, and mesothelial cells. No atypical cells were seen.

The dog was hospitalized for 2 days for observation and was treated with clopidogrel (Plavix, Bristol‐Myers Squibb, Bridgewater, New Jersey) 4 mg/kg PO q24h to be continued long term. At the time of discharge from hospital, both the gross abdominal distension and severity of SC edema appeared significantly improved. The dog was reexamined 1 week after discharge and both the abdominal distension and SC edema had resolved and the dog was reported to be well at home. Repeat echocardiography at this point showed a subjective reduction in the size of the ventricular thrombus. Repeat physical examination after 3 weeks detected no abnormalities and the thrombus could no longer be visualized on echocardiogram. There were no echocardiographic changes suggestive of pulmonary hypertension.

Blood drawn by jugular venipuncture at 2 and 6 weeks after initial presentation was submitted to a commercial human laboratory to investigate the possibility of a congenital thombophilic disease. Measurement of prothrombin and activated‐partial thromboplastin times were repeated at both time points and results were within normal limits on both occasions. The dog was clinically well at the timing of both samples. Both platelet‐poor plasma and serum were submitted. For preparation of platelet‐poor plasma, blood was initially collected into 3.2% sodium citrate tubes, which were centrifuged at 2000*g* for 15 minutes. The citrated plasma was then pipetted from the citrated tubes into plain conical tubes, taking care to avoid the plasma‐cell interface. The citrated plasma was respun for a further 15 minutes, and the plasma was then transferred into a plain tube, taking care to avoid aspiration of any residual cells in the conical part of the tube. Serum was prepared by centrifuging whole blood at 3000*g* for 10 minutes. Samples were frozen at −20°C within 30 minutes of sampling. Samples were transported to the laboratory, frozen overnight, and all samples were analyzed within 1 week. Measurement of protein C activity by Chromogenix Coamatic chromogenic assay (Quadratech Diagnostics, Lewes, UK) on a Sysmex CS2000i automated analyzer (Sysmex UK, Milton Keynes, UK) was markedly reduced at both 2 and 6 weeks after presentation being 47.5 and 8.7 iu/dL, respectively (canine reference range 75%‐135%). Phenotypic assays for antithrombin activity and activated protein C resistance were normal with reference to canine normal ranges in the literature for same‐type assays. An immunoturbidimetric antigenic assay for free protein S and ELISA for protein C failed to provide readings as the captured antibodies did not cross‐react with canine proteins. Antiphospholipid antibodies have been reported in dogs,^7^ but persistence of lupus anticoagulant, anticardiolipin antibodies, anti‐β2 glycoprotein antibodies, or antiprothrombin antibodies was not demonstrated in this case.

The dog was diagnosed with a congenital protein C deficiency leading to thrombus formation. Administration of the previously prescribed clopidogrel was stopped and administration of the selective direct factor Xa inhibitor, rivaroxaban (Xarelto, Janssen Pharmaceuticals, Titusville, New Jersey) 1 mg/kg PO q24h, was started. The dog remains clinically well at home with approximately 6 months follow‐up.

## 3 DISCUSSION

This is a report describing a congenital protein C deficiency in a dog leading to clinical signs associated with thrombosis. Based on the history and physical examination findings in this case, a disease‐causing venous congestion from the abdomen and abdominal wall was initially suspected as the cause for the clinical signs. Unexpectedly, the thrombus was detected within the right ventricle and the authors suspect that this had recently embolized from the caudal vena cava and previous vascular obstruction due to this thrombus was the cause for the reported clinical signs. The rapid resolution of clinical signs without specific intervention would support this hypothesis. On consideration of diseases known or suspected to result in a hypercoagulable state in dogs, a predisposing cause for thrombus formation had not been identified during initial investigations. Bile‐acid simulation testing returned normal results and the presence of a portosystemic shunt vessel was excluded based on the results of the CT. There was no evidence of a concurrent inflammatory state and the dog did not fulfill the requirements for making a diagnosis of disseminated intravascular coagulopathy. This was the reason for pursuing investigation of conditions known to predispose to thrombosis in human patients.

A diagnosis of congenital protein C deficiency is made in human patients upon documenting a persistent decrease in plasma protein C concentrations in the absence of any of the previously mentioned acquired causes of deficiency.^8^ Testing at the time of acute thrombosis is not recommended in people. It has been shown that measurements of protein C concentration made on samples taken within 24 hours of presentation for a thrombotic event can be unreliable for making a diagnosis of congenital deficiency in a small number of patients because of consumption.^9^, ^10^ Repeat testing is recommended to confirm persistence, and deficiency of protein C should be confirmed on more than 1 sample before making the diagnosis.^10^ More than 270 different mutations of the protein C gene have been documented in people.^8^ While genetic testing can prove useful in certain cases to differentiate congenital from acquired protein C deficiency (where the possibility of an acquired cause cannot be excluded), genetic testing might be most useful to assist with family counseling, antenatal diagnosis, and making a definitive diagnosis in severely affected neonates.^11^, ^12^

Chromogenic assays are recommended for initial evaluation of protein C deficiency in humans because interferences that falsely prolong or shorten clotting times in clot‐based assays cannot operate in chromogenic assays whose analytical principles are independent of coagulation pathways.^10^ A canine‐specific assay for measurement of protein C is not available but adaption of a human chromogenic assay has been validated for use in dogs.^13^ Published reference values for protein C activity in dogs, measured with functional chromogenic assays developed for use in humans, have been established at 75%‐135%.^5^, ^6^, ^7^, ^13^, ^14^ A chromogenic assay with the same analytic principles as those previously published in dogs was used to measure plasma protein C activity in this case. Protein C is stable in both human and canine samples for at least 2 weeks when stored frozen at −20°C.^13^ In this case, samples taken at 2 and 6 weeks after diagnosis showed a persistent and marked decrease in plasma protein C concentrations and in the absence of any conditions known to cause an acquired protein C deficiency, the findings are consistent with a congenital deficiency. Genetic testing or measurement of plasma protein C activity in the parents or another first‐degree relative could not be performed.

Current recommendations from the American College of Chest Physicians endorse the use of anticoagulant medication for an indefinite period in patients suffering an unprovoked venous thromboembolic event.^15^ In the absence of prolonged anticoagulant use, recurrent thrombotic events occur in 30% of patients with a hereditary thrombophilia within 5 years of an unprovoked venous thrombus.^16^ Direct oral anticoagulants (DOACs) are commonly used to treat hereditary thrombophilias in human patients to reduce the risk of recurrent thrombosis. A recent review of the use of direct oral anticoagulants in humans has shown positive results in patients with hereditary thrombophilic disease.^17^ Rivaroxaban is a DOAC, which inhibits factor Xa, and its use has been described in human patients with congenital protein C deficiency.^18^ Rivaroxaban has in vitro and in vivo anticoagulant effects in dogs.^19^, ^20^ Its use has been described in the clinical setting in dogs with confirmed thromboembolic disease and for prophylaxis in dogs with immune‐mediated hemolytic anemia.^21^, ^22^ In this case, administration of the previously prescribed clopidogrel was stopped, and administration of rivaroxaban was started upon the finding of protein C deficiency and based on the use of this drug for thromboprophylaxis in affected human patients. Measurement of prothrombin time was scheduled at 3 monthly intervals ongoing to monitor the effect of rivaroxaban administration. Achieving a 1.5‐1.9 times delay of prothrombin time from baseline might be a practical method for therapeutic monitoring of dogs receiving rivaroxaban.^23^

The clinical signs associated with thrombosis resolved relatively quickly in this case and the dog remains well at home 6 months after initial presentation with no clinical signs suggestive of recurrent thrombosis. The dog is to remain on daily rivaroxaban indefinitely. Congenital thrombophilic disease should be considered in dogs with thrombosis for which an underlying disease classically thought to predispose to a hypercoagulable state cannot be found. Long‐term treatment with DOACs is likely appropriate.

## CONFLICT OF INTEREST DECLARATION

Gary Moore was employed by the commercial laboratory at which the testing for potential thrombophilic diseases was performed.

## OFF‐LABEL ANTIMICROBIAL DECLARATION

Authors declare no off‐label use of antimicrobials.

## INSTITUTIONAL ANIMAL CARE AND USE COMMITTEE (IACUC) OR OTHER APPROVAL DECLARATION

Authors declare no IACUC or other approval was needed.

## HUMAN ETHICS APPROVAL DECLARATION

Authors declare human ethics approval was not needed for this study.
